# Quantum coherent tractor beam effect for atoms trapped near a nanowaveguide

**DOI:** 10.1038/srep28905

**Published:** 2016-07-21

**Authors:** Mark Sadgrove, Sandro Wimberger, Síle Nic Chormaic

**Affiliations:** 1 Research Institute of Electrical Communications, Tohoku University, Katahira 2-1-1, Aoba-ku, Sendai-shi Japan; 2 Institut für Theoretische Physik, Universität Heidelberg, Philosophenweg 12, 69120 Heidelberg, Germany; 3 Dipartimento di Fisica e Scienze della Terra, Universitádi Parma, Via G. P. Usberti 7/a, 43124 Parma, Italy; 4 INFN, Sezione di Milano Bicocca, Gruppo Collegato di Parma, Italy; 5 Light-Matter Interactions Unit, Okinawa Institute of Science and Technology Graduate University, Onna-son, Okinawa 904-0495, Japan

## Abstract

We propose several schemes to realize a tractor beam effect for ultracold atoms in the vicinity of a few-mode nanowaveguide. Atoms trapped near the waveguide are transported in a direction *opposite* to the guided mode propagation direction. We analyse three specific examples for ultracold ^23^Na atoms trapped near a specific nanowaveguide (i.e. an optical nanofibre): (i) a conveyor belt-type tractor beam effect, (ii) an accelerator tractor beam effect, and (iii) a quantum coherent tractor beam effect, all of which can effectively pull atoms along the nanofibre toward the light source. This technique provides a new tool for controlling the motion of particles near nanowaveguides with potential applications in the study of particle transport and binding as well as atom interferometry.

Optical tractor beams, where light incident from a single direction causes particles to move against the light propagation direction and towards the beam source, are an intriguing example of science fiction^1^ inspiring the creation of a scientific technology. Along with the intrinsic interest of realizing such a counterintuitive transport phenomenon, such beams can be useful for simplifying experimental geometries, or creating pulling forces where a light intensity gradient (such as that required for optical tweezers) cannot be readily achieved. Optical tractor beams have been realized very recently in a number of experiments^2^, which may be divided into two categories: (a) Those where dissipative effects, including scattering forces^3^ and photophoretic effects^4^ are required and (b) those where conservative forces trap and move particles, for example, using overlapping co-propagating beams^5^,^6^. Schemes using cold atoms and hollow-core fibres^7^, which guide light that is red-detuned from atomic resonance, could also be considered as tractor-beam effects within category (b), although the limited particle capture position (restricted to the end of the fibre) along with the limited capture range (due to large beam divergence at the fibre end) are major points of difference with the other tractor beam effects considered here.

Here, we show that tractor beam effects of type (b) may also be realized in a nanowaveguide setting. Indeed, more complicated schemes are feasible as compared to free space, due to the fact that field confinement occurs over an arbitrary distance, irrespective of wavelength. We also introduce an additional category of tractor beam effect (c) in which only conservative forces are used but no axial confinement of atoms is required allowing atomic spatial coherence to be maintained. We call this a *quantum coherent tractor beam effect*.

The basic principle used to realize optical tractor beams using conservative potentials is the superposition of two copropagating electromagnetic fields. Assuming for simplicity the two fields have the same polarization and amplitude *E*_0_/2, the general form of the superposed fields is


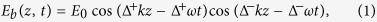


where Δ^±^*k* = (*k*_2_ ± *k*_1_)/2, Δ^±^*ω* = (*ω*_2_ ± *ω*_1_)/2 and *k*_*i*_ and *ω*_*i*_ are the wavenumber and angular frequency of field *i* for *i* ∈ {1, 2}. The field is a travelling wave with spatial and temporal frequencies given by the respective mean of the two incident fields’ spatial and temporal frequencies. This travelling wave is amplitude modulated by the beat between the two fields. We will assume that the *k*_*i*_ and *ω*_*i*_ can be chosen independently, and focus on the case where *ω*_1_ = *ω*_2_ = *ω*. (Although we will introduce small detunings between *ω*_1_ and *ω*_2_ to realise the tractor beam effect, this condition will always be met to a good approximation). In this case, the field is given by


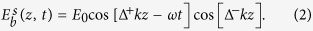


This field shares an important property with electromagnetic standing waves, namely nodes and antinodes with fixed spatial positions decided by the time independent beat envelope cos (Δ^−^*kz*). However, we note that for co-propagating waves, the condition *ω*_1_ = *ω*_2_ is not trivial to satisfy in free space when *k*_1_ and *k*_2_ differ. At this point, the advantage of using few-mode nanowaveguides can be seen: atoms can interact with the evanescent region of the guided modes, and a standing beat field can be generated using two copropagating modes of the waveguide. It is precisely this principle which we will use to achieve a tractor beam effect. Note that from now on, we drop the superscript *s* from 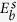 with the understanding that all beating fields considered in this paper will be standing beat fields of the form given in Eq. 2.

Now consider a two level atom subject to the field *E*_*b*_. As in the case of an optical standing wave, the potential experienced by the atom has a spatial dependence with period given by the envelope of the field. Specifically, we have


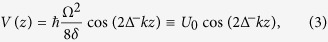


where Ω = 2*μE*_*b*_/ħ is the Rabi frequency of the atom in the field, with *μ* the atomic electrical dipole moment, and *δ* is the detuning from atomic resonance.

We delineate two regimes of interest: One where *U*_0_ is large compared to the atomic kinetic energy *V*_*k*_, and the other where *U*_0_ is of the same order or smaller than *V*_*k*_. In the first regime, the potential traps atoms at the sites of maximum or minimum intensity (for *δ* > 0 or *δ* < 0 respectively) and individual atoms are localised at these sites. Then *U*_0_ is refered to as the trap depth, and can be written in a more convenient form


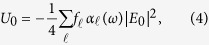


where *f*_*ℓ*_ is the absorption oscillator strength of the atomic transition under consideration, and *α*_*ℓ*_(*ω*) is the real part of the atomic polarizability for the transition at a frequency *ω*. *α*_*ℓ*_ is given by^8^,^9^


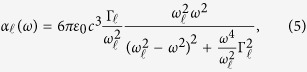


where *ε*_0_ is the vacuum electric permeability, *c* is the speed of light, and 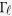 and *ω*_*ℓ*_ are the decay rate and frequency of the atomic transition respectively. We can find the photon scattering rate using the formula 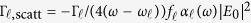.

In what follows, we will consider ^23^Na atoms prepared in the 3S ground state. In this case, only the two transitions associated with the D_1_ and D_2_ lines have a significant strength^10^. Labelling these two transitions by *ℓ* = 1 and *ℓ* = 2 respectively, we have *f*_1_ = 0.320, Γ_1_ = 61.353 × 10^6^ s^−1^, and *λ*_1_ = 589.756 nm, and *f*_2_ = 0.640, Γ_2_ = 61.542 × 10^6^ s^−1^, *λ*_2_ = 589.158 nm^11^. We ignore the hyperfine structure of the atomic transitions due to the large detuning. We also ignore magnetic sublevels which we assume to be initially equally populated. Our decision to use Na rather than Cs or Rb which are more commonly used in optical nanowaveguide based experiments with cold atoms^12^,^13^,^14^,^15^,^16^,^17^, is due to its lower atomic mass which gives favourable scaling of the atomic recoil velocity for the resonant Bragg transition we will consider later in the paper. However, the principles considered here hold for any atomic species which can be optically trapped near to a nanowaveguide.

In the second regime of interest, where *U*_0_ ≤ *V*_*k*_, the potential may additionally be pulsed on with some pulse width *T*_*p*_. In this case, atoms are not trapped by the potential, and in general the atomic wavefunction is modulated by the presence of the field and the amount of modulation is governed by the so-called kick strength^18^


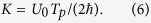


We will make use of both of the above mentioned regimes in the remainder of the paper.

## Results

### Scheme (i): Conveyor belt tractor beam effect

In the following, we will focus on a specific type of nanowaveguide - a vacuum-clad silica nanofibre, known as an optical nanofibre. The guided modes of an optical fibre have an electric field of the form **E** = **E**(*r*, *θ*, *z*)exp[−i(*βz* − *ω*_*L*_*t*)]. For a given angular frequency *ω*_*L*_, and a fixed fibre radius *a*, a finite number of modes exist with different values of the propagation constant *β*. In order for tractor beam effects to be realized on an optical nanofibre, the fundamental mode, and at least one higher order mode must be supported in the nanofibre region. We note that recent experiments have demonstrated such few-mode optical nanofibres^14^,^19^,^20^.

**Figure 1:**
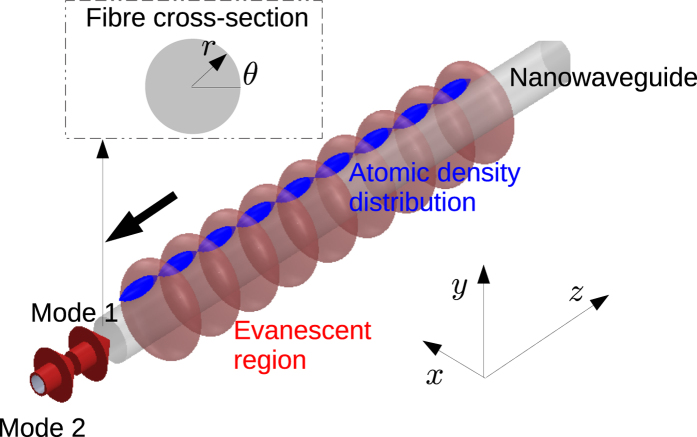
Conceptual diagram. Two guided modes of a nanowaveguide beat to form a periodic, evanescent field near its surface. Atoms may be trapped in the field and moved towards the light source (negative *z* direction) using one of the three techniques detailed in the text.

In scheme (i), we wish to establish the simplest scheme for an experimentally feasible tractor beam effect. A conceptual diagram of the scheme is shown in Fig. 1. Note that here and throughout the paper, we use cartesian coordinates with axes as shown in Fig. 1 and polar coordinates as indicated in the inset of Fig. 1. This scheme uses two different techniques which are already well known - dual mode, blue detuned trapping^21^,^22^ and the optical conveyor belt technique^23^, which has been experimentally demonstrated for optical nanofibres^24^. Note that the details of this scheme are substantially similar to Sagué *et al*.^22^. There are two points of difference: Substitution of Na here for Cs, and the introduction of a frequency detuning between the two modes. Our new contribution is to point out that such a scheme can support a tractor beam effect which has not been noted before to the best of our knowledge.

The nanofibre total guided electric field has the form


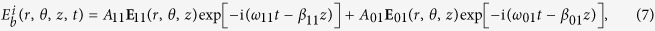


where *A*_11_ and *A*_01_ are the amplitudes of the HE_11_ mode function, **E**_11_, and the TE_01_ mode function, **E**_01_, respectively. The calculation of the mode amplitudes from the desired optical power in each mode along with the mode function definitions is found in the Appendix. We take *ω*_01_ = *ω*_11_ + *δ*_*c*_, and require 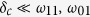. In this case, the trapping potential experienced by atoms in the beating field is given by


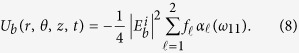


We set the diameter of the optical nanofibre to 430 nm and take *λ* = 588 nm. The two mode trap is created by the interference of the quasi *y*-polarized HE_11_ mode of the nanofibre with the TE_01_ mode. We set an experimentally realistic power limit of 50 mW for the total optical power in the optical nanofibre, and require a trapping depth of at least 500 *μ*K and a trapping position at least 200 nm from the optical nanofibre surface. We also require that the trap lifetime be at least 1 s, which is two orders of magnitude greater than the expected time required for the proposed experimental sequence. This leads to the choice of 3.47 mW and 0.47 mW for the optical powers in the HE_11_ and TE_01_ modes, respectively. For these parameters, we find propagation constants of *β*_11_ = 1.05 × 10^7 ^m^−1^ for HE_11_ and *β*_01_ = 0.91 × 10^7 ^m^−1^ for TE_01_.

**Figure 2:**
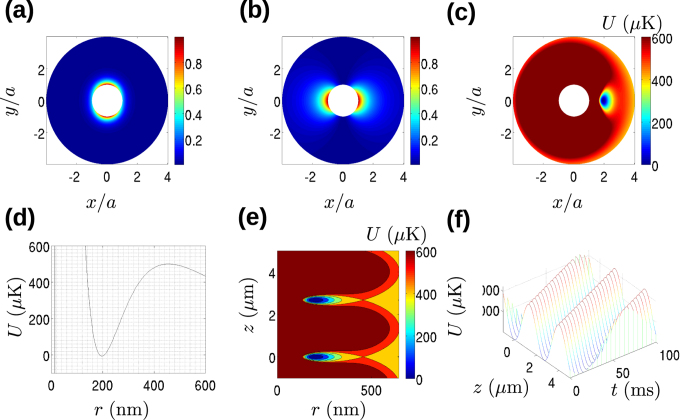
Conveyor belt tractor beam scheme. (**a**,**b**) Normalized intensities in the *x* − *y* plane and outside the nanofibre for the HE_11_ mode and TE_01_ mode, respectively. (**c**) The trapping potential in the *x* − *y* plane experienced by ^23^Na atoms due to the superposed modes. (**d**) Radial dependence of the two-mode trap potential at *θ* = 0. (**e**) Axial variation of the trapping potential on the right hand side of the nanofibre. (**f**) Demonstration of the tractor beam effect showing the periodic trapping potential moving towards the light source as a function of time.

Normalized intensities of the *y*-polarized components of the HE_11_ and TE_01_ modes (which interfere to create the trap) are shown in Fig. 2(a,b), respectively. Superposition of the two modes creates the trapping potential seen in Fig. 2(c). The radial and azimuthal trapping frequencies are 5.0 MHz and 3.4 MHz, respectively, as found by fitting a harmonic approximation to the potential about the trap centre. It has been shown that the side of the nanofibre on which the trap is located for *z* = 0 depends on the relative phase between the two modes, with trapping potentials alternating between the left and right side once each optical period^22^. We will consider just the right side of the nanofibre (i.e. *θ* = 0) and assume a relative phase of zero between the two nanofibre modes in what follows without loss of generality. The radial trapping potential is shown in Fig. 2(d). Here, and in schemes (ii) and (iii) below, we reference *r* = 0 to the nanofibre surface when plotting the radial potential. Additionally, here and for the radial potentials used in schemes (ii) and (iii) below, the effect of the van der Waals potential near the nanofibre surface is included using the so-called “flat” approximation *U*_vdW_ = −*C*_3_/(*r* − *a*)^3^ which slightly overestimates the van der Waals potential away from the nanofibre surface^8^,^22^. We estimated the value of *C*_3_ to be 3.2 × 10^−49 ^Jm^3 ^ 8. This value is smaller than the value of 5.6 × 10^−49^ Jm^3^ for Cs atoms and bulk silica, suggesting that our choice of sodium should not be disadvantageous compared to the more commonly used Cs when it comes to atom losses due to the effect of the nanofibre surface.

For the purposes of developing our concept in schemes i) and ii), we will assume that atoms of temperature ~10 *μ*K can be introduced to the trap without significant losses or heating. Note that in typical experiments in this field where a magneto-optical trap is overlapped with an optical nanofibre, heating due to collisions with the nanofibre typically causes significantly larger temperatures^25^,^26^. We will consider the issues of loading and atom temperature in more detail in scheme iii). For atoms with a temerature of 10 *μ*K, the maximum scattering rate in the trap is Γ_scatt_ = 6.4 s^−1^. This scattering rate corresponds to a trap lifetime of *t*_life_ = *T*_trap_/(*T*_rec_ ∗ Γ_scatt_) = 33 s, where *T*_trap_ ≈ 500 *μ*K is the radial trap depth, and *T*_rec_ = 2.4 *μ*K is the recoil temperature for ^23^Na.

Under the conditions listed above, atoms will be trapped in a lattice along each side of the nanofibre at the *θ* = 0 and *θ* = *π*. The case for *θ* = 0 (right hand side of the fibre) is shown in Fig. 2(e). The period of the lattice of traps is 2.7 *μ*m and the axial trapping frequency at the trap centre is 2 kHz. To create a tractor beam effect, it is simply necessary to make the lattice move towards the light source using the so-called optical conveyor belt effect. This can be achieved by detuning the TE_01_ mode relative to the HE_11_ mode. A detuning of *δ*_*c*_ results in a velocity of


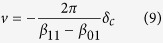


of the trapping lattice along the *z*-axis. Figure 2(f) shows the case for a detuning *δ*_*c*_ = 10 kHz, resulting in trapped atoms moving at a velocity of 0.027 ms^−1^. Note that despite the movement of the trapping lattice, since *δ* ≪ *ω* we are still in the standing beat regime to a good approximation.

### Scheme (ii): Accelerator tractor beam effect

Scheme (i) as outlined above is the simplest tractor beam effect achievable using a nanowaveguide, requiring as it does just two modes. However, the scheme localizes atoms axially which reduces their possible applications in situations requiring atomic spatial coherence (i.e. matter wave interference effects). As a first step towards liberating the spatial coherence of atoms in the tractor beam, we now consider a more complicated scheme than (i). As before, atoms are moved towards the light source using a blue detuned, two-mode trapping array. However, in the present scheme, this trapping lattice is superimposed on a two-colour trap. Once atoms have been accelerated by moving the lattice, the lattice can be switched off diabatically leaving the atoms travelling along the waveguide towards the light source at a mean velocity equal to the final velocity of the trapping lattice.

We first consider the two-colour trap. A two-colour nanofibre trap is formed by combining two fundamental modes in a nanofibre, one red detuned and one blue detuned from atomic resonance^8^,^27^,^28^. Following recent experimental implementations^29^,^30^, we choose the blue detuned, HE_11_ mode, 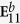
to be polarized orthogonally to the red detuned, HE_11_ mode, 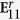 where the superscripts *r* and *b* denote the red detuned and blue detuned modes. The potentials due to each mode separately are 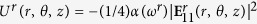 and 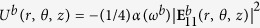, where *ω*^*r*^ and *ω*^*b*^ are the red and blue detuned field frequencies, respectively. Assuming a large frequency difference between the red and blue detuned modes, the total trapping potential may be found by simply adding the red and blue trapping potentials: *U*_2col_ = *U*^*r*^ + *U*^*b*^.

To achieve the maximum trap depth we require the potentials to be equal at the surface of the nanofibre, i.e. *U*^*r*^(*r* = *a*, 0, 0) = *U*^*b*^(*r* = *a*, 0, 0)^8^. The radial trap position may then be chosen by adjusting the relative power in the red and blue detuned modes. Finally, the mode powers can be adjusted while preserving their relative ratio in order to choose the trap depth while preserving the radial trap position.

**Figure 3:**
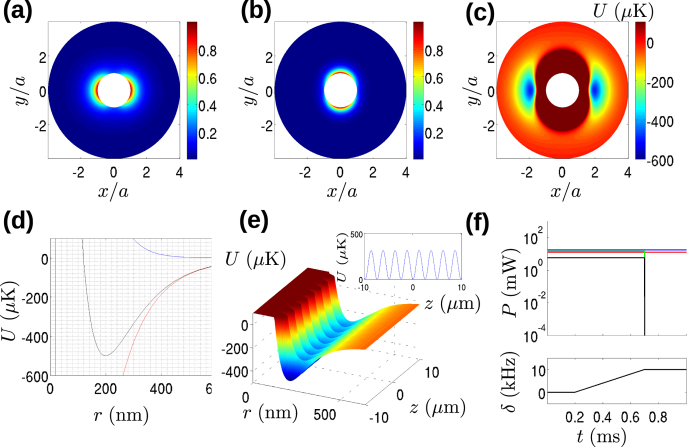
Accelerator tractor beam scheme. (**a**,**b**) Normalized intensities in the *x* − *y* plane for the red and blue detuned HE_11_ modes of the two-colour trap, respectively. (**c**) Two-colour trapping potential in the *x* − *y* plane and outside the nanofibre surface. (**d**) Radial dependence of the two-colour trapping potential at *θ* = 0. (**e**) The lattice potential superposed on the two-colour trap. The inset shows the axial trapping potential at the radial trap minimum. (**f**) Sequence for realizing the accelerator tractor beam effect. The upper panel shows the powers for the red-detuned 2 colour trapping field HE_11_ mode (red line), blue-detuned 2-colour trapping field HE_11_ mode (blue line), the HE_11_ mode of the trapping lattice field (green line) and the TE_01_ line of the trapping lattice (black line). The lower panel shows the detuning of the trapping lattice TE_01_ mode.

We take the nanofibre radius to be the same as in scheme i). In the present example, we set wavelengths *λ*^*r*^ = 850 nm, *λ*^*b*^ = 570 nm, and powers *P*^*r*^ = 12.7 mW and *P*^*b*^ = 17.3 mW. Normalized mode intensity profiles for the red and blue detuned modes are shown in Fig. 3(a) (red) and (b) (blue). The trapping potential over the nanofibre cross-section is shown in Fig. 3(c). Figure 3(d) shows the radial dependence of the potential demonstrating that the trap depth is 500 *μ*K, and the trap minimum position is 200 nm from the nanofibre surface. The radial trap frequency is 3.8 MHz and the azimuthal trap frequency is 1.7 MHz. The potential has no *z* dependence, and it therefore defines a waveguide for atoms on either side of the nanofibre.

Next, we add a trapping lattice by superimposing a two-mode, blue detuned potential of the same form as that used in scheme i). This time, we choose a wavelength *λ*_*b*_ = 500 which is different to that used to create the potentials *U*^*b*^ and *U*^*r*^ so that the beat frequency between all of the different wavelength fields is much higher than the characteristic frequency of the atomic dynamics and may be ignored^7^. We then choose the optical power of the two modes to create as deep a trap as possible within the 50 mW total power limit. Specifically, we choose a power of 14.3 mW for the HE_11_ mode and 5.7 mW for the TE_01_ mode. Superimposing the beat potential *U*_*b*_ for these parameters on the two-colour trap potential produces the total potential *U* = *U*_2col_ + *U*_*b*_ which has the form shown in Fig. 3(e). The inset of Fig. 3(e) shows the axial trapping potential at the minimum of the radial trapping potential. It may be seen that a trapping lattice of depth ~300 *μ*K and lattice period Λ = 2.56 *μ*m is formed on top of the two-colour trap. The axial frequency of the trapping lattice is *f*_ax_ = 433 kHz. We also note that the electric field which creates the trapping lattice is *y* polarized for positions sufficiently close to the *x*-axis, due to the vanishing *z* polarization of the *y* polarized HE_11_ mode at *θ* = 0. Because the two-colour trap confines atoms within ~±10 degrees of the *x*-axis, we will assume that atoms experience a *y*-polarized lattice potential.

The scattering rate at the trap centre for the two-colour trap is 25 s^−1^ corresponding to a trap lifetime of about 8 s. In the presence of the trapping lattice, the lifetime reduces to about 1.5 s. However, as shown below, the trapping lattice is only applied for 1 ms, and so it has little influence on the overall atom loss in the present scheme.

In order to propel atoms towards the light source, the experimental sequence shown in Fig. 3(f) is applied. During an initial loading time (the exact time is unspecified here and would be determined experimentally to give maximum loading efficiency), atoms are introduced into the trap with all fields on and no detuning between the modes of the two mode trap. At the end of the loading time, chosen arbitrarily to be 200 *μ*s for this example, the detuning of the TE_01_ mode is increased from 0 to 10 kHz over 500 *μ*s leading to a final speed of *v*_*f*_ = 0.025 ms^−1^. The acceleration of the atoms is 49 ms^−2^, much less than the maximum axial acceleration in the trapping lattice which is of order 
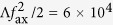
 ms^−2^ and so the sweep may be considered to be adiabatic. After the sweep finishes at time *t* = 700 *μ*s, the power of the trapping lattice modes is rapidly reduced to 0 (experimentally achievable switching rates are typically of order 100 ns using an acousto-optic modulator) and atoms move with constant velocity in the two-colour trap induced waveguide towards the light sources at a mean velocity *v*_*f*_.

### Scheme (iii): Quantum coherent tractor beam effect

In scheme (ii) described above, we presented calculations for a moving trapping lattice superimposed on a two-colour trap which could be used to accelerate atoms towards the light source. Although the atoms may propagate freely down the waveguide after the trapping lattice is turned off, the confinement of atoms to individual optical lattice sites reduces the spatial extent of the atomic wavepackets, making the use of atomic spatial coherence, e.g. for interferometric purposes, difficult. In order to develop a tractor beam scheme which can liberate the spatial coherence of the atoms, we need a way to exert a directional force on the centre-of-mass of the atomic wavepacket without confining the atoms axially.

We now propose just such a scheme which makes use of an atomic wavepacket beam-splitting technique known as a Bragg pulse in which a moving periodic potential resonantly couples atoms in the motional ground state |*p* = 0〉 of the lattice potential to a higher motional eigenstate of the lattice |*p* = *nħk*〉, where *p* is the atomic momentum, *n* is an integer and *k* is the lattice wavenumber. We note from the outset that this scheme makes assumptions about the atomic initial state and its subsequent motion which are experimentally challenging, and we comment on this point in the discussion. In particular we will assume that (a) atoms with a temperature of about 10 nK can be generated initially sufficiently far from the nanofibre to avoid heating and (b) that they can subsequently be adiabatically loaded into the two-colour trap without significant extra heating. We also assume that (c) the atomic gas is dilute enough that the atoms may be considered to be in the non-interacting regime. Finally we assume that (d) the motion of atoms along the fibre axis can be treated independently of their transverse motion. These strict assumptions allow us to build a very simple model of a spatially coherent tractor beam effect which we detail below.

In the case of *n* = 1, a *π*/2 Bragg pulse creates a momentum space superposition state 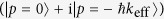
31. Atoms in this state can then be accelerated towards the light source using the so-called *resonance ratchet effect*^31^,^32^,^33^. This effect may be understood heuristically in terms of the spatial atomic wavefunction as follows: The Bragg pulse imprints a sinusoidal density profile on the atomic wavepacket due to the AC Stark effect. The wavepacket can then be “kicked” predominantly in the negative (positive) direction by applying a second optical potential with the same spatial period, but with a phase *ϕ* such that the high density part of the wavepacket is aligned with the negative (positive) gradient of the kicking potential. Furthermore, kicking pulses may be applied whenever the atomic wavepacket rephases which happens at multiples of the Talbot time *T*_*T*_ = 4*π*/*ω*_*r*_^34^,^35^,^36^. Applying kicks at each Talbot time is known as the quantum resonance condition, and hence this phenomenon is often named the “resonance ratchet effect”.

Although this effect is best understood in terms of the spatial wavefunction, calculations are simplest when expressed in momentum space. The final state associated with this process has a momentum space wavefunction *ψ*_*f*_ = 〈*m*|*ψ*_*i*_〉 given by^31^


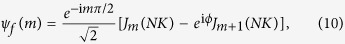


where *m* labels the momentum space eigenstates of the lattice, *J*_*m*_ are Bessel functions of the first kind, and *N* is the number of kicks applied. The associated momentum space probability distribution is


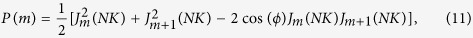


and the mean momentum of an atom in the state may be shown to be −*NK*/2, i.e., the atom is accelerated along the nanofibre towards the light source. Such a scheme can clearly be regarded as a tractor beam effect, but unlike all previous tractor beams to the best of our knowledge, it both requires and preserves quantum spatial coherence. In what follows, we will restrict ourselves to the case where *N* = 1, i.e. a single kick immediately follows the Bragg pulse. This is because of the dephasing effects during the “free evolution” in the two-colour trap waveguide which will spoil the perfect rephasing necessary for the Talbot effect to occur. These dephasing effects arise from differential light shifts experienced by atoms in the two-colour trap due to both radial and azimuthal variations of the light shift in the trap and vector-light shift effects due to the circular polarization of the two-colour trap^37^.

**Figure 4:**
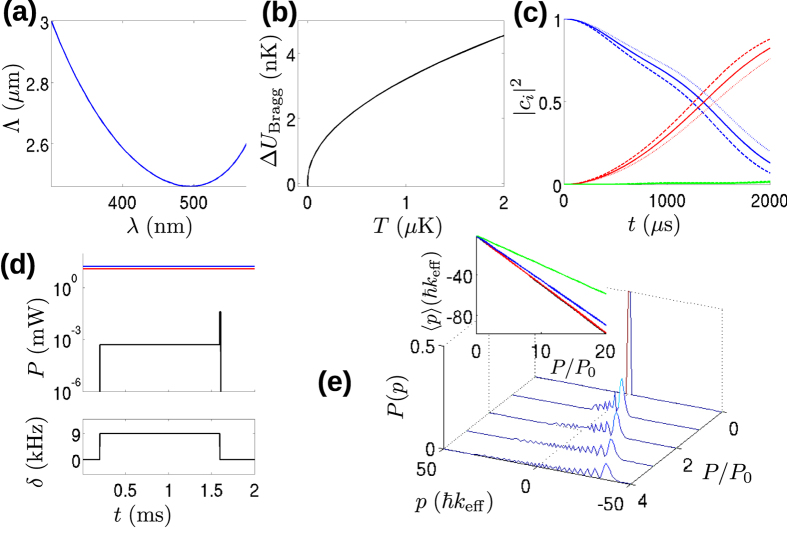
Quantum coherent tractor beam scheme. (**a**) Dependence of the lattice constant Λ on the guided mode wavelength *λ* for the HE_11_ and TE_01_ superposition used to create the lattice potential. (**b**) Dependence of the maximum variation Δ*U*_Bragg_ of the Bragg pulse potential experienced by atoms in the two-colour trap as a function of the atomic temperature *T*. (**c**) Evolution of the squared amplitudes |*c*_*i*_|^2^ for the 0 momentum component (*i* = 0, blue line), *ħk*_eff_ momentum component (*i* = 1, red line) and 2*ħk*_eff_ (*i* = 2, red line) components of the atomic wavefunction as a function of the Bragg pulse time *t*. The solid lines show the result for an atom at the centre of the trap and the dashed and dotted lines show the result for the maximum and minimum Bragg potential respectively experienced by an atom with an effective temperature of 1 *μ*K. (**d**) Experimental sequence used to realize the quantum coherent tractor beam effect. The upper panel shows the powers for the red-detuned 2 colour trapping field HE_11_ mode (red line), blue-detuned 2-colour trapping field HE_11_ mode (blue line), and the HE_11_ and TE_01_ modes of the trapping lattice field (black line). The lower panel shows the detuning of the trapping lattice TE_01_ mode. (**e**) The momentum distribution of an atom after the Bragg pulse and a kick have been applied shown for several values of *P*/*P*_0_. The inset shows how the mean momentum 〈*p*〉 depends on the power in the kick for the case of |*c*_0_|^2^ = |*c*_1_|^2^ = 0.5 (black line), |*c*_0_|^2^ = 0.56, |*c*_1_|^2^ = 0.44 (red line, case corresponding to the dashed and dotted lines in (**c**)), |*c*_0_|^2^ = 0.7, |*c*_1_|^2^ = 0.3 (blue line) and |*c*_0_|^2^ = 0.9, |*c*_1_|^2^ = 0.1 (green line).

We now give the parameters for the quantum coherent tractor beam effect. The two-colour trap parameters are the same as in scheme (ii). To achieve an efficient Bragg pulse and reduce both the Bragg pulse time and the Talbot time, we wish to reduce the lattice constant Λ as much as possible for the given nanofibre radius *a* = 215 nm. As shown in Fig. 4(a), a clear minimum in the value of Λ occurs for a wavelength of approximately 500 nm. This minimum occurs due to the competing influence of the falling wavelength (which reduces Λ), and the associated falling penetration depth into the vacuum which reduces the difference in the propagation constant between the HE_11_ and TE_01_ modes (which increases Λ). We therefore choose 500 nm for the wavelength of the two modes used to create the optical lattice potential as in scheme ii). We introduce HE_11_ and TE_01_ modes at *λ*_*b*_ = 500 nm and powers of 500 nW each in addition to the two-colour trap. These parameters produce an axial lattice potential of trap depth *U*_Bragg_ = 18 nK and with lattice period Λ = 2.46 *μ*m. The first order Bragg frequency for this lattice constant is *f*_*B*_ = 9.0 kHz.

Because scheme (iii) relies on coherence between the atomic wavepacket and the periodic potential created by the Bragg pulse, it is necessary to ask whether dephasing will ruin the tractor beam effect. The most likely cause of such dephasing in the simple model under assumptions (a) – (d) is the radial inhomogeneity of the lattice potential depth caused by the exponential decay of the fields away from the fibre surface. If atoms have a significant velocity component in the radial direction, then they will experience different potential depths and thus different coupling to the *ħk*_eff_ momentum state during the Bragg pulse. Such an effective temperature in the radial direction might result from imperfect adiabatic loading of nK temperature atoms into the nanofibre trap. The maximum variation of the Bragg pulse potential depth Δ*U*_Bragg_ as a function of atom temperature *T* is shown in Fig. 4(b).

Using a standard method^38^ (see the Methods section for details), and ignoring light shifts of sublevels of the atomic ground state which are negligible compared with the detuning of the fields which create the lattice potential, we simulated the effect of a Bragg pulse for these parameters leading to the results shown in Fig. 4(c), which show that a *π*/2 pulse takes 1303 *μ*s. We also simulated Bragg pulses for atoms experiencing potentials corresponding to the maximum and minimum values assuming movement to either side of the trap centre for an effective atomic temperature of 1 *μ*K. The results are shown by the dashed and dotted lines in Fig. 4(d). For the nominal Bragg *π*/2 pulse time of 1303 *μ*s, the maximum radial displacement of an atom towards the nanofibre gives weights of |*c*_0_|^2^ = 0.44 for the |*p* = 0〉 component and |*c*_1_|^2^ = 0.56 for |*p* = *ħk*_eff_〉 component. For maximum radial displacement away from the nanofibre, the results are approximately reversed, i.e., |*c*_0_|^2^ = 0.56 for the |*p* = 0〉 component and |*c*_1_|^2^ = 0.44 for the |*p* = 0〉 and |*p* = −*ħk*_eff_〉 component.

The experimental sequence for the quantum coherent tractor beam effect is shown in Fig. 4(d). First, atoms with an initial momentum spread Δ*p* < *ħk*_eff_ are loaded into the two-colour trap. After loading, the detuning of the TE_01_ mode relative to the HE_11_ mode is set to *δ*_*c*_ = *f*_*B*_, and the power in both modes is raised to 500 nW for 1303 *μ*s to create a *π*/2 Bragg pulse. Immediately after the Bragg pulse, the detuning between the lattice beams is set to *δ*_*c*_ = 0 and the optical power in each mode of the lattice potential light is raised to *P*_0_ = 42 *μ*W and a pulse of width 10 *μs* is applied to the atoms. These pulse parameters give a kick strength *K* ≈ 10 accelerating the mean atomic momentum by (*K*/2)*ħk*_eff_. After the kick, the mean atomic velocity is 0.035 ms^−1^ towards the light source (i.e. in the negative *z* direction). This velocity can be increased by increasing the power *P* of the kicking field as shown in Fig. 4(e). The inset of Fig. 4(e) shows the mean momentum for several different weightings of the |*p* = 0〉 and |*p* = −*ħk*_eff_〉 states as a function of *P*/*P*_0_. We note that for the variation in Bragg pulse potentials experienced by atoms with a radial temperature of 1 *μ*K, there is no discernible difference in the tractor beam effect. Indeed even for weightings as different as |*c*_0_|^2^ = 0.9 and |*c*_1_|^2^ = 0.1, a significant momentum current remains, suggesting that the tractor beam effect should be robust against dephasing caused by *μ*K scale atomic temperatures in the radial direction.

## Discussion

We now discuss notable aspects of the quantum spatially coherent scheme (iii). The tools required to realize scheme (iii) for atoms trapped near an optical nanofibre are the same as for scheme (ii), and our calculations demonstrate that the required optical potential can be achieved within typical experimental parameters. However, we wish to note that with regard to achieving the required atomic initial state, along with the subsequent one-dimensional evolution of the atom in the two-colour trap, the assumptions (a) to (d) which we used to arrive at our simple model have yet to be investigated theoretically or experimentally.

Regarding assumptions (a) and (b) in scheme (iii), in order for the quantum coherent scheme to transport atoms efficiently, the atoms must have a small initial momentum spread along the *z* axis relative to the lattice momentum spacing *ħk*_eff_. In experiments involving atoms in free space, this has typically been achieved by using atoms sourced from a Bose-Einstein condensate (BEC), although use of a velocity selective transition to produce atoms with a narrow initial momentum distribution is also a possibility. We note that loading of atoms from a BEC into a nanofibre based optical trap, or otherwise producing atoms with a narrow axial momentum distribution in a nanofibre trap has not yet been realized experimentally and may pose a significant challenge. Nonetheless, the recent realization of BECs within hundreds of nm of a nano-optical device^39^ leads us to believe that it is experimentally feasible. It may also be possible to create BECs near to nanofibres using all optical techniques where the lensing effect of the nanofibre itself could provide a dimple trap^40^ suitable for efficient, all optical evaporation to BEC. We also note the relatively long period of the lattice considered here (Λ ~ 2 *μ*m) as compared to free space standing wave experiments (*λ* ~ 500 nm). The equivalent temperature for the momentum quantum *ħk*_eff_ of the lattice used here is 17 nK. While the temperature of atoms sourced from BECs is typically between 10 and 100 nK, we note that ^23^Na BECs with temperatures as low as 450 pK have been achieved^41^, and so the requirement that the momentum spread Δ*p* be much less than *ħk*_eff_ is certainly experimentally feasible even for the lattice periods considered in scheme (iii). Recent progress in optical lattice experiments in effective one dimensional BECs where the transverse trapping frequencies are much higher than the axial ones, and radial trap sizes may be smaller than 100 nm^42^, as in scheme (iii) lead us to believe that these assumptions are not unreasonable. Nonetheless, the long de Broglie wavelengths associated with these low temperatures is expected to make loading atoms into the nanofibre trap challenging. A fully three dimensional hydrodynamic simulation can clarify the best strategy for the loading of ultracold atoms into the proposed trap, but this is beyond the scope of the present study.

On the other hand, if sufficiently low temperature atoms are hard to prepare, the effect of the finite temperature of the atomic sample on the directed transport effect considered here has recently been discussed elsewhere^43^. Furthermore, we note that spatially quantum coherent ratchet schemes using thermal atoms do exist and could be used in place of the resonance ratchet effect to achieve a similar result to scheme (iii)^44^. However, in such schemes the role of quantum spatial coherence is not as clear as in the present proposal.

Moving on to possible issues regarding the few mode optical nanofibre, one possible difficulty in the realization of all of the schemes discussed above is the issue of coupling selectively to the TE_01_ mode rather than the TM_01_ mode since both have very similar propagation constants at nanofibre diameters close to *λ*/2. However, the inadvertent coupling of some of the input power to the TM_01_ mode is expected to have a minimal effect on the schemes discussed here due to the orthogonality of the TE_01_ and TM_01_ modes. This means that the presence of small amounts of the TM_01_ mode will tend to offset the total trap potential energy without having a large effect on the trap shape or depth. Additional concerns regarding the stability of trapping parameters to changes in optical power in the two mode and two-colour trapping schemes considered here have already been dealt with in the literature^22^,^29^,^30^. As an additional note, the taper down to the nanofibre region of the optical nanofibre must be designed so that it is adiabatic for all wavelengths used in the experiment. In practice, we might expect different transmissions through the taper for the different wavelengths used, but these can be measured and allowed for in the experiment. This issue presents no obstacle to the scheme presented here in principle, and the necessary control over higher order modes in a nanofibre has already been demonstrated in proof of principle experiments^14^,^19^,^20^.

Given the above challenges, we emphasize that the valuable points of our present study are to introduce the concept of tractor beam effects on nanowaveguides in imminently experimentally realizable situations (schemes i and ii) and to produce a simple model of a spatially quantum coherent tractor beam effect using a nanowaveguide (scheme iii), where there is more freedom to vary parameters (i.e. via the waveguide index and its spatial modes) than equivalent free space schemes using, e.g., Bessel beams. The exact implementation which best allows the realization of such a scheme is a question for future detailed study.

Finally, we comment on the utility of our scheme and the new possibilities opened up by realizing tractor beams on nanowaveguides. Our focus above on optical nanofibres raises the question of how useful a tractor beam effect is in the nanowaveguide setting. After all, unlike in free space, a counterpropagating beam can be introduced into either side of a nanofibre merely by introducing light into that side’s connecting fibre, the end of which can be arbitrarily located. This would allow a standard optical lattice to be formed, and thus standard transport techniques to be used. This objection, however, ignores the fact that a number of recent studies of cold atoms near nanowaveguides used one-sided coupling to a waveguide^45^,^46^, making it very difficult to introduce a counterpropagating beam. Nonetheless, atoms near such a nanowaveguide surface could be moved along the waveguide axis using any of the schemes i) through iii) described here. We also note that some proposals for nanofibre – cold atom interfaces use single sided fibre tapers^47^.

As for properties of our tractor beam proposals that present new possibilities compared with standard free space realizations, perhaps the most intriguing is the possibility of pulling objects along curved paths. It has been shown that the trapping schemes considered here are robust under bends in the nanofibre with radii of order 10 *μ*m^48^. This fact adds an entirely new flexibility to tractor beam effects which is impossible to realize in free space. Additionally, nanowaveguides have advantages over free space when it comes to realizing the spatially quantum coherent tractor beam effect of scheme (iii). This is because the lattice period may be decreased in nanowaveguides by increasing the refractive index of the waveguide material. This should allow the realization of Bragg pulses using standing beat potentials at rates comparable with those achievable in counterpropagating standing wave configurations. As we have noted above, scheme (iii) is unique amongst tractor beam effects that we are aware of since it both requires and preserves quantum spatial coherence. To our knowledge, this makes the first tractor beam effect which can be used in situations where wavepacket interference is required (e.g. in atom interferometry applications). We also note that this spatially quantum coherent scheme occupies a new regime for tractor beams in which neither dissipative scattering or longitudinal confinement of particles is used. Instead, the quantum coherence between the wave packet and the lattice potential allow a directional force to be exerted on the atomic wavepacket.

We anticipate that the theoretical demonstration of a new platform for optical tractor beam effects using nanowaveguides along with the identification of a new class of quantum coherent tractor beam effects as given here will provide a fruitful starting point for new studies of this intriguing and useful transport phenomenon.

## Methods

### Atom in a standing beat electric field

The interaction picture Hamiltonian for an atom exposed to the field *E*_*b*_ can be derived by following the prescription of Graham *et al*. as laid out for an atom of mass *M* in an optical standing wave in ref. 49. First, we write the total Hamiltonian for the atom in the field using the dipole and rotating wave approximations:


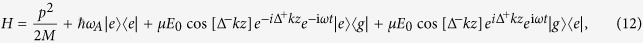


where *p* and *z* are the respective one dimensional momentum and position operators along the nanofibre axis, *e* and *g* label the excited and ground states of the atom respectively, *ω*_*a*_ is the angular atomic resonance frequency, and *μ* is the atomic dipole moment. The parameters *E*_0_, *ω* and Δ^±^*k* are as defined in the main text.

Following standard procedures^49^, we convert to the interaction picture to remove the time dependence, and the Hamiltonian becomes


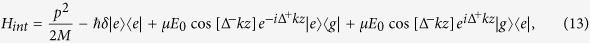


where *δ* = *ω* − *ω*_*a*_. Finally, we write the general atomic state as |Ψ〉 = *ψ*_*g*_(*z*, *t*)|*g*〉 + *ψ*_*e*_(*z*, *t*)|*e*〉, and assume large *δ* allowing us to adiabatically eliminate the excited state to give the ground state Schrödinger equation


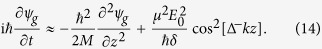


Note that the factors 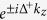 are canceled in the adiabatic elimination step. We note that a natural time scale in this system is given by the recoil frequency *ω*_*r*_ = *ħ*(Δ^−^*k*)^2^/(2*M*_Na_), where *M*_Na_ is the atomic mass of ^23^Na.

Equivalently, we may write the Hamiltonian of the atomic ground state as


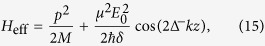


where we have ignored a constant energy offset between Eqs 14 and 15. If we turn on the periodic potential for only a short time 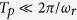, then atoms receive a brief momentum “kick”. The strength of the kick is usually quantified by the dimensionless parameter 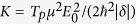.

This Hamiltonian is identical to that for an atom in a standing wave formed by counterpropagating beams of wavenumber Δ^−^*k*_*z*_. Therefore, in principle any dynamics which can be realized for an atom in a (pulsed) standing wave can also be realized for an atom in the field *E*_*b*_.

### Fibre mode functions

#### HE_11_ mode

The fundamental mode of optical nanofibres is the HE_11_ hybrid mode in which all six components of the electromagnetic field are non-zero. For the purposes of the present research, we restrict our attention to the electric field lying outside the nanofibre surface. For a fibre of radius *a*, with core refractive index *n*_co_ and cladding refractive index *n*_cl_, the eigenvalue equation which allows the propagation constant *β* to be found is^50^


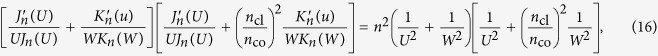


where 
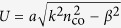
 and 
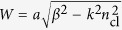
. The mode function in cylindrical coordinates is given by


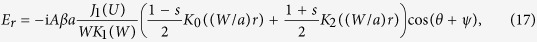



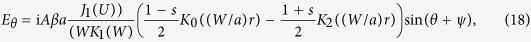



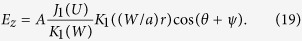


for *r* > *a* where *J*_*n*_(*x*) is a Bessel function of the first kind and *K*_*n*_(*x*) is a modified Bessel function of the second kind. The parameter *s* is given by


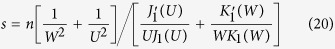


Additionally we note that two degenerate polarizations are defined by the above equations - the *x* polarization denoted 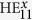 where *ψ* = 0 and the *y* polarization denoted 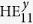 where *ψ* = *π*/2.

For a given optical power, *P*, the mode amplitude *A* can be calculated from the propagating power using the following equations:


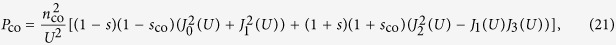



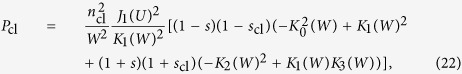



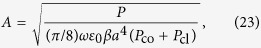


where 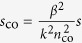, and 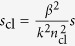. *P*_co_ and *P*_cl_ are the optical powers in the fibre core and cladding regions respectively.

#### TE_01_ mode

In the case of the TE_01_ mode, the eigenvalue equation is considerably simpler than that for the fundamental mode:


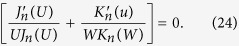


The electric field outside the fibre is given by:


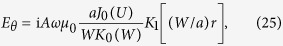


with all other components 0. The equation relating the amplitude *A* to a given optical power *P* is


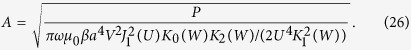


### Calculation of the Van der Waals coefficient *C*_3_

The optical nanofibre trapping potentials considered in this paper are all modified by the van der Waals potential due to the nanofibre surface. Here, we approximate this potential by that due to a bulk silica surface. This potential has the form


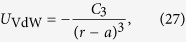


where the coefficient *C*_3_ is specific to the combination of the atomic species and the material of the surface. In the case of ^23^Na atoms near the surface of bulk silica, which is applicable to the examples studied in this paper, *C*_3_ may be calculated as shown below. For the definition of polarizability *α* used in this paper, we find^8^


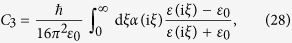


where *ε*(*ω*) is the dielectric function of silica at frequency *ω*. The dielectric function of silica may be expressed as^8^


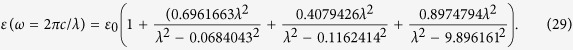


For *α*(*ω*), we use the sum of the expression for the D_1_ and D_2_ lines of ^23^Na, as given in Eq. 5.

For completeness, we note that an alternative approximation for *α* which can be used to calculate *C*_3_ is given by^51^


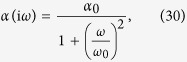


where *α*_0_ = 24.1 Å^3^ is the DC polarizability of sodium^51^. Calculating *C*_3_ using this expression for polarizability uses a more commonly found form of Eq. 28 due to Lifshitz:


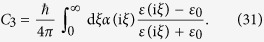


We evaluated both Eqs 28 and 31 numerically and found the same result *C*_3_ = 3.2 × 10^−49^ Jm^3^ by both methods. We caution the reader, however, that some commercial software packages do not numerically evaluate the integrals 28 and 31 successfully. We used Mathematica to achieve the given result.

### Simulation of the Bragg pulse

In the following simulation of the effect of the Bragg pulse, we assume that different light shifts in ground state sublevels have a negligible effect on the dynamics of atoms during the pulse due to the large detuning of the field which creates the lattice potential. First, we Bloch decompose the atomic wavepacket giving^38^


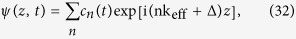


where the *c*_*n*_ are the amplitudes of the states |*p* = *nħk*_eff_〉, and *ħ*Δ is the effective momentum offset created by the movement of the potential due to the frequency difference between the two modes. For the first order Bragg transition, this frequency is *f*_*B*_ = 9 kHz and Δ = *k*_eff_. Note that, as mentioned in the body of the paper, *k*_eff_ is the wave number of the lattice itself, not that of a running wave, as is typical in treatments of atoms in standing wave potentials. Substituting Eq. 32 into the Schrödinger equation gives^38^


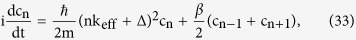


where *β* = *U*_Bragg_/*ħ*.

We numerically solve this set of coupled differential equations on a truncated basis of 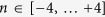 starting from the state with *c*_0_ = 1. The truncated basis is allowed because of the resonant coupling due to the Bragg pulse which leads to very little amplitude outside the *p* = 0 and *p* = *ħk*_eff_ states as may be seen in Fig. 4(d). The required time for a Bragg *π*/2 pulse as used in the quantum coherent tractor beam effect may be found by solving the equations up to a time *t*_Bragg_ when |*c*_0_|^2^ = |*c*_1_|^2^. We note that we have used this simulation method elsewhere in investigating related phenomena for kicked atoms^52^.

### Simulation of the kick

We now consider the effect of a pulse from a periodic optical potential (a “kick”) on the atomic wavepacket. If the pulse is short (relative to 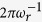), as in this work, we can use the *δ*-kick approximation, in which the atom is assumed not to move during the kick. In this case, the evolution operator for the atom can be approximated by *U*_kick_ = exp(i*K* cos (2Δ^−^*kz*)) (i.e. the contribution of free evolution can be ignored during the kick). For an atom in an initial momentum eigenstate of the potential |*n*〉, the momentum space wavefunction over the eigenstates |*m*〉 afer the kick can be shown to be *ψ* = 〈*m*|*U*_kick_|*n*〉 = i^*n*−*m*^*J*_*m*−*n*_(*K*)^18^, where *J*_*l*_ is a first order Bessel function of order *l*. Using this identity, along with the initial state of the atom after the Bragg pulse, we arrive at Eq. 10 for the state of the atom after the kick.

## Additional Information

**How to cite this article**: Sadgrove, M. *et al*. Quantum coherent tractor beam effect for atoms trapped near a nanowaveguide. *Sci. Rep.* **6**, 28905; doi: 10.1038/srep28905 (2016).

## References

[b1] Krauss L. M. The Physics of Star Trek (Basic Books, 2007).

[b2] Sáenz J. J. Optical forces: Laser tractor beams. Nat. Photonics 5, 514–515 (2011).

[b3] Chen J., Ng J., Lin Z. & Chan C. T. Optical pulling force. Nat. Photonics 5, 531–534 (2011).

[b4] Shvedov V., Davoyan A. R., Hnatovsky C., Engheta N. & Krolikowski W. A long-range polarization-controlled optical tractor beam. Nat. Photonics 8, 846–850 (2014).

[b5] Brzobohaty O. . Experimental demonstration of optical transport, sorting and self-arrangement using a ‘tractor beam’. Nat. Photonics 7, 123–127 (2013).

[b6] Ruffner D. B. & Grier D. G. Optical Conveyors: A Class of Active Tractor Beams. Phys. Rev. Lett. 109, 163903 (2012). 23215079 10.1103/PhysRevLett.109.163903

[b7] Renn M. J. . Laser-Guided Atoms in Hollow-Core Optical Fibers. Phys. Rev. Lett. 75, 3253–3256 (1995). 10059537 10.1103/PhysRevLett.75.3253

[b8] Le Kien F., Balykin V. I. & Hakuta K. Atom trap and waveguide using a two-color evanescent light field around a subwavelength-diameter optical fiber. Phys. Rev. A 70, 063403 (2004).

[b9] Grimm R., Weidemüller M. & Ovchinnikov Y. B. Optical dipole traps for neutral atoms. Adv. At. Mol. Opt. Phys. 42, 95–170 (2000).

[b10] Sansonetti J. E., Martin W. C. & Young S. L. Handbook of Basic Atomic Spectroscopic Data, *Natl. Inst. Stand. Technol.*, Gaithersburg, MD, http://physics.nist.gov/Handbook.

[b11] Steck D. A. Sodium D Line Data. Available online at http://steck.us/alkalidata.

[b12] Nayak K. P. . Optical nanofiber as an efficient tool for manipulating and probing atomic fluorescence. Optics Express 15, 5431–5438 (2007). 19532797 10.1364/oe.15.005431

[b13] Sagué G., Vetsch E., Alt W., Meschede D. & Rauschenbeutel A. Cold-Atom Physics Using Ultrathin Optical Fibers: Light-Induced Dipole Forces and Surface Interactions. Phys. Rev. Lett. 99, 163602 (2007). 17995250 10.1103/PhysRevLett.99.163602

[b14] Kumar R. . Interaction of laser-cooled ^87^Rb atoms with higher order modes of an optical nanofibre. New J. Phys. 17, 013026 (2015).

[b15] Kato S. & Aoki T. Strong Coupling between a Trapped Single Atom and an All-Fiber Cavity. Phys. Rev. Lett. 115, 093603 (2015). 26371652 10.1103/PhysRevLett.115.093603

[b16] Béguin J.-B. . Generation and Detection of a Sub-Poissonian Atom Number Distribution in a One-Dimensional Optical Lattice. Phys. Rev. Lett. 113, 263603 (2014). 25615331 10.1103/PhysRevLett.113.263603

[b17] Nieddu T., Gokhroo V. & Nic Chormaic S. Optical nanofibres and neutral atoms. J. Opt. 18, 053001 (2016).

[b18] Oberthaler M. K., Godun R. M., d’Arcy M. B., Summy G. S. & Burnett K. Observation of Quantum Accelerator Modes. Phys. Rev. Lett. 83, 4447–44450 (1999).

[b19] Frawley M. C., Petcu-Colan A., Truong V. G. & Nic Chormaic S. Higher order mode propagation in an optical nanofiber. Opt. Communications 285, 4648–4654 (2012).

[b20] Hoffman J. E., Fatemi F. K., Beadie G., Rolston S. L. & Orozco L. A. Rayleigh scattering in an optical nanofiber as a probe of higher-order mode propagation. Optica 2, 416 (2015).

[b21] Fu J., Yin X. & Tong L. Two-colour atom guide and 1D optical lattice using evanescent fields of high-order transverse modes. J. Phys. B: At. Mol. Opt. Phys. 40, 4195 (2007).

[b22] Sagué G., Baade A. & Rauschenbeutel A. Blue-detuned evanescent field surface traps for neutral atoms based on mode interference in ultrathin optical fibres. New J. Phys. 10, 113008 (2008).

[b23] Schrader D. . An optical conveyor belt for single neutral atoms. Appl. Phys. B 73, 819–824 (2001).

[b24] Schneeweiss P. . A nanofiber-based optical conveyor belt for cold atoms. Appl. Phys. B 110, 279–283 (2013).

[b25] Russell L., Deasy K., Daly M. J., Morrissey M. J. & Nic Chormaic S. Sub-Doppler temperature measurements of laser-cooled atoms using optical nanofibres. Meas. Sci. Technol. 23, 015201 (2012).

[b26] Grover J. A., Solano P., Orozco L. A. & Rolston S. L. Phys. Rev. A 92, 013850 (2015).

[b27] Le Kien F., Balykin V. I. & Hakuta K. State-Insensitive Trapping and Guiding of Cesium Atoms Using a Two-Color Evanescent Field around a Subwavelength-Diameter Fiber. J. Phys. Soc. Japan 74, 910–917 (2005).

[b28] Fu J., Yin X., Li N. & Tong L. Atom waveguide and 1D optical lattice using a two-color evanescent light field around an optical micro/nano-fiber. Chinese Opt. Lett. 6, 112–115 (2008).

[b29] Vetsch E. . Optical Interface Created by Laser-Cooled Atoms Trapped in the Evanescent Field Surrounding an Optical Nanofiber. Phys. Rev. Lett. 104, 203603 (2010). 20867028 10.1103/PhysRevLett.104.203603

[b30] Goban A. . Demonstration of a State-Insensitive, Compensated Nanofiber Trap. Phys. Rev. Lett. 109, 033603 (2012). 22861848 10.1103/PhysRevLett.109.033603

[b31] Sadgrove M., Horikoshi M., Sekimura T. & Nakagawa K. Rectified Momentum Transport for a Kicked Bose-Einstein Condensate. Phys. Rev. Lett. 99, 043002 (2007). 17678359 10.1103/PhysRevLett.99.043002

[b32] Dana I., Ramareddy V., Talukdar I. & Summy G. S. Experimental Realization of Quantum-Resonance Ratchets at Arbitrary Quasimomenta. Phys. Rev. Lett. 100, 024103 (2008). 18232872 10.1103/PhysRevLett.100.024103

[b33] Salger T. . Directed transport of atoms in a Hamiltonian quantum ratchet. Science 326, 1241 (2009). 19965469 10.1126/science.1179546

[b34] Ryu C. . High-Order Quantum Resonances Observed in a Periodically Kicked Bose-Einstein Condensate. Phys. Rev. Lett. 96, 160403 (2006). 16712208 10.1103/PhysRevLett.96.160403

[b35] Deng L. . Temporal, Matter-Wave-Dispersion Talbot Effect. Phys. Rev. Lett. 83, 5407–5410 (1999).

[b36] Sadgrove M. & Wimberger S. A pseudo-classical method for the atom-optics kicked rotor: from theory to experiment and back. Adv. At. Mol. Opt. Phys. 60, 315 (2011).

[b37] Le Kien F., Schneewies P. & Rauschenbeutel A. Dynamical polarizability of atoms in arbitrary light fields: general theory and application to cesium. Eur. Phys. J. D 67, 92 (2013).

[b38] Hughes K. J., Burke J. H. T. & Sackett C. A. Suspension of Atoms Using Optical Pulses, and Application to Gravimetry. Phys. Rev. Lett. 102, 150403 (2009). 19518607 10.1103/PhysRevLett.102.150403

[b39] Stehle C. . Plasmonically tailored micropotentials for ultracold atoms. Nat. Photonics 5, 494 (2011).

[b40] Le Kien F. & Hakuta K. Microtraps for atoms outside a fiber illuminated perpendicular to its axis: Numerical results. Phys. Rev. A 80, 013415 (2009).

[b41] Leanhardt A. E. . Cooling Bose-Einstein Condensates Below 500 Picokelvin. Science 301, 1513 (2003). 12970559 10.1126/science.1088827

[b42] Moritz H., Söferle T., Köhl M. & Esslinger T. Exciting collective oscillations in a trapped 1D gas. Phys. Rev. Lett. 91, 250402 (2003). 14754099 10.1103/PhysRevLett.91.250402

[b43] Summy G. & Wimberger S. Quantum random walk of a Bose-Einstein condensate in momentum space. Phys. Rev. A 93, 023638 (2016). 10.1103/PhysRevLett.121.070402 30169047

[b44] Jones P. H., Goonasekera M., Meacher D. R., Jonckheere T. & Monteiro T. S. Directed Motion for Delta-Kicked Atoms with Broken Symmetries: Comparison between Theory and Experiment. Phys. Rev. Lett. 98 073002 (2007). 17359021 10.1103/PhysRevLett.98.073002

[b45] Thompson J. D. . Coupling a single trapped atom to a nanoscale optical cavity. Science 340, 1202 (2013). 23618764 10.1126/science.1237125

[b46] Tiecke T. G. . Nanophotonic quantum phase switch with a single atom. Nature 508, 241 (2014). 24717513 10.1038/nature13188

[b47] Kato S., Chonan S. & Aoki T. High-numerical-aperture microlensed tip on an air-clad optical fiber. Opt. Lett. 39, 773–776 (2014). 24562203 10.1364/OL.39.000773

[b48] Barnett A. H. . Substrate-based atom waveguide using guided two-color evanescent light fields. Phys. Rev. A 61, 023608 (2000).

[b49] Graham R., Schlautmann M. & Zoller P. Dynamical localization of atomic-beam deflection by a modulated standing light wave. Phys. Rev. A 45, R19 (1992). 9906767 10.1103/physreva.45.r19

[b50] Okamoto K. Fundamentals of optical waveguides. Academic Press (2006).

[b51] Perreault J. D., Cronin A. D. & Savas T. A. Using atomic diffraction of Na from material gratings to measure atom-surface interactions. Phys. Rev. A 71, 053612 (2005).

[b52] Sadgrove M., Wimberger S. & Nakagawa K. Phase-selected momentum transport in ultra-cold atoms. Eur. Phys. J. D 66, 155 (2012).

